# Supplementary material for: The Evolution and Characterization of the RNA Interference Pathways in Lophotrochozoa
Source: Genome Biol Evol. 2024 May 7;16(5):evae098. doi: 10.1093/gbe/evae098 (PMC11114477; doi:10.1093/gbe/evae098)
Supplement: evae098_Supplementary_Data [file evae098_supplementary_data.zip › Supplementary_materials.zip!/selected_miRNAs/NoGe_selected_miRNAs/mirdeep_pdf_files/NMRB01002279.1_Notospermus_geniculatus_strain_Ushimado_scaffold2279,_whole_genome_shotgun_sequence_37126.pdf]

Provisional ID	:	NMRB01002279.1_Notospermus_geniculatus_strain_Ushimado_scaffold2279,_whole_genome_shotgun_sequence_37126
Score total	:	113444.2
Score for star read(s)	:	3.9
Score for read counts	:	113435.7
Score for mfe	:	1.6
Score for randfold	:	0
Score for cons. seed	:	3
Total read count	:	222511
Mature read count	:	222441
Loop read count	:	0
Star read count	:	70

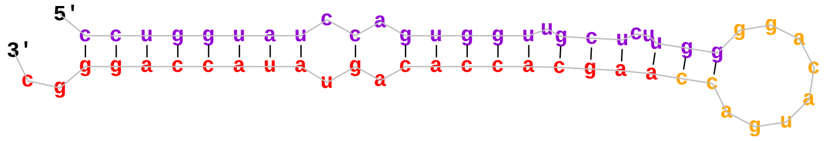

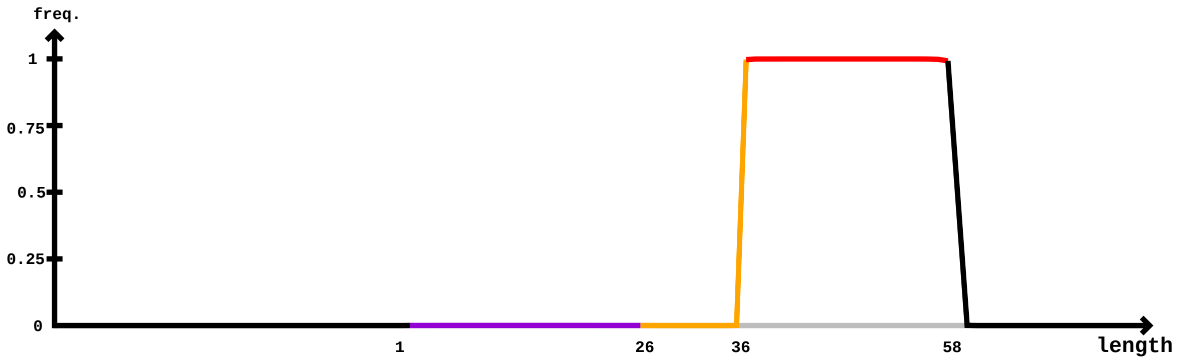

Star **Mature**

```
5'- uaucagauaauggagaaaauugcauggcuuuguccccugguauccagugguugcucuuggggacaugaccaagcaccacaguauaccagggcauaugcaugcuucuagauuc   -3'    obs
    uaucagauaauggagaaaauugcauggcuuuguccccugguauccagugguugcucuuggggacaugaccaagcaccacaguauaccagggcauaugcaugcuucuagauuc          exp
    .....((...(((((......(((((....(((.(((((((((.(.(((((.(((..(((........))))))))))).).))))))))).)))..))))))))))...))   reads   mm   sample
    ...................................ccugguauccagugguugcucu.......................................................   1       0    Mm3
    ...................................ccugguauccagugguugcucuug.....................................................   1       0    Mm3
    ...................................ccugguauccagugguugcucuugg....................................................   9       0    Mm3
    ...................................ccugguaAccagugguugcucuugg....................................................   1       1    Mm3
    ...................................ccugguauccagugguugcucuuggA...................................................   3       1    Mm3
    ...................................ccugguauccagugguugcucuuggggacaugaccU.........................................   1       1    Mm3
    .....................................................................caagcaccacaguauaccaggg.....................   3       0    Mm3
    .....................................................................caagcaccacaguauaccagggc....................   16      0    Mm3
    .....................................................................caagcaccacaguauaccagggcU...................   1       1    Mm3
    .....................................................................caagcaccacaguauaccagggca...................   1       0    Mm3
    ......................................................................aagcaccacaguauaccUg.......................   1       1    Mm3
    ......................................................................aagcaccacaguauaccag.......................   2       0    Mm3
    ......................................................................aagcaccacaguauaccagg......................   30      0    Mm3
    ......................................................................aagcaccacaguauaccaggC.....................   2       1    Mm3
    ......................................................................aagcaccacaguauaccaggU.....................   1       1    Mm3
    ......................................................................aagcaccacaguauaccGggg.....................   1       1    Mm3
    ......................................................................aagcaccacaguUuaccaggg.....................   1       1    Mm3
    ......................................................................aagcaccacaguauacAaggg.....................   1       1    Mm3
    ......................................................................aagcaccacaguauaccCggg.....................   2       1    Mm3
    ......................................................................aagcaccacaguauaccaggg.....................   159     0    Mm3
    ......................................................................aagcaccacaguauaccaggA.....................   2       1    Mm3
    ......................................................................aagcaccacaguauaccagAgc....................   8       1    Mm3
    ......................................................................aagcaccacaguauaccaggAc....................   5       1    Mm3
    ......................................................................aagcaUcacaguauaccagggc....................   6       1    Mm3
    ......................................................................Uagcaccacaguauaccagggc....................   14      1    Mm3
    ......................................................................Cagcaccacaguauaccagggc....................   2       1    Mm3
    ......................................................................aGgcaccacaguauaccagggc....................   6       1    Mm3
    ......................................................................aCgcaccacaguauaccagggc....................   3       1    Mm3
    ......................................................................aagcCccacaguauaccagggc....................   3       1    Mm3
    ......................................................................aagcaccacaguaAaccagggc....................   4       1    Mm3
    ......................................................................aagcaccacaguauaccagggc....................   29157   0    Mm3
    ......................................................................aagcaccacUguauaccagggc....................   4       1    Mm3
    ......................................................................aagcaccacaguauaccCgggc....................   107     1    Mm3
```

Star **Mature**

```
uaucagauaauggagaaaauugcauggcuuuguccccugguauccagugguugcucuuggggacaugaccaagcaccacaguauaccagggcauaugcaugcuucuagauuc
......................................................................aagcaccacaguauaccaggUc....................   7    1   Mm3
......................................................................aagcaccaUaguauaccagggc....................   16   1   Mm3
......................................................................aagcaccGcaguauaccagggc....................   5    1   Mm3
......................................................................aagcUccacaguauaccagggc....................   7    1   Mm3
......................................................................aagcaccacaguauGccagggc....................   13   1   Mm3
......................................................................aagcacUacaguauaccagggc....................   15   1   Mm3
......................................................................aagcaccacaguaCaccagggc....................   4    1   Mm3
......................................................................aagcaccacaguauUccagggc....................   3    1   Mm3
......................................................................aaCcaccacaguauaccagggc....................   1    1   Mm3
......................................................................aaUcaccacaguauaccagggc....................   5    1   Mm3
......................................................................aagcaccacaguUuaccagggc....................   5    1   Mm3
......................................................................aagcaccacaguaGaccagggc....................   3    1   Mm3
......................................................................aagcaccacagAauaccagggc....................   2    1   Mm3
......................................................................Nagcaccacaguauaccagggc....................   18   1   Mm3
......................................................................aagcaccacaguauaccagggA....................   22   1   Mm3
......................................................................aagcaccacaguauaccGgggc....................   71   1   Mm3
......................................................................aagcaccacaguauaccaggCc....................   15   1   Mm3
......................................................................aagcaccacaCuauaccagggc....................   35   1   Mm3
......................................................................aagcaccacaguauaccagggU....................   55   1   Mm3
......................................................................aagcaccacaAuauaccagggc....................   40   1   Mm3
......................................................................aagcaGcacaguauaccagggc....................   2    1   Mm3
......................................................................aagcaccacaguauacUagggc....................   97   1   Mm3
......................................................................aagGaccacaguauaccagggc....................   2    1   Mm3
......................................................................aagcacAacaguauaccagggc....................   1    1   Mm3
......................................................................aagcaccacGguauaccagggc....................   23   1   Mm3
......................................................................Gagcaccacaguauaccagggc....................   8    1   Mm3
......................................................................aagcaccacaguauaccagUgc....................   6    1   Mm3
......................................................................aagcaccacCguauaccagggc....................   1    1   Mm3
......................................................................aagcaccacaUuauaccagggc....................   20   1   Mm3
......................................................................aagcaccacaguGuaccagggc....................   4    1   Mm3
......................................................................aagcaccCcaguauaccagggc....................   5    1   Mm3
......................................................................aagcaccacaguauaccaUggc....................   4    1   Mm3
......................................................................aaAcaccacaguauaccagggc....................   7    1   Mm3
......................................................................aagcaccacaguauaccUgggc....................   94   1   Mm3
......................................................................aagcaNcacaguauaccagggc....................   4    1   Mm3
......................................................................aagcaccacaguauaUcagggc....................   4    1   Mm3
......................................................................aagcacNacaguauaccagggc....................   3    1   Mm3
......................................................................aagcaAcacaguauaccagggc....................   2    1   Mm3
......................................................................aagcaccacaguauacAagggc....................   145  1   Mm3
......................................................................aagcaccacaguauaccaAggc....................   3    1   Mm3
......................................................................aagcaccacaguauaccagCgc....................   1    1   Mm3
......................................................................aagcaccacaguauaccaCggc....................   1    1   Mm3
......................................................................aagcaccacaguauacGagggc....................   37   1   Mm3
......................................................................aUgcaccacaguauaccagggc....................   4    1   Mm3
......................................................................aagcGccacaguauaccagggc....................   3    1   Mm3
......................................................................aagcaccUcaguauaccagggc....................   6    1   Mm3
......................................................................aagcaccacagCauaccagggc....................   4    1   Mm3
......................................................................aagcaccacaguauaccagggG....................   4    1   Mm3
......................................................................aagUaccacaguauaccagggc....................   5    1   Mm3
......................................................................aagcaccacaguauCccagggc....................   1    1   Mm3
......................................................................aagcaccacaguauaccagggcU...................   528  1   Mm3
......................................................................aagcaccacaguauaccagggcG...................   9    1   Mm3
......................................................................aagcaccacaguauaccagggcC...................   14   1   Mm3
......................................................................aagcaccacaguauaccagggca...................   54   0   Mm3
......................................................................aagcaccacaguauaccagggcUu..................   14   1   Mm3
......................................................................aagcaccaGaguauaccagggcau..................   4    1   Mm3
......................................................................aagcaccacaguauaccagggcaG..................   1    1   Mm3
......................................................................aagcaccacaguauaccagggcaA..................   1    1   Mm3
......................................................................aagcaccacaguauaccagggcau..................   4    0   Mm3
......................................................................aagcaccacaguauaccagggcCu..................   3    1   Mm3
......................................................................aagcaccaGaguauaccagggcaua.................   1    1   Mm3
......................................................................aagcaccacaguauaccagggcauU.................   1    1   Mm3
......................................................................aagcaccacaguauaccagggcaAa.................   1    1   Mm3
.......................................................................agGaccacaguauaccagggc....................   1    1   Mm3
.......................................................................aUcaccacaguauaccagggc....................   1    1   Mm3
.......................................................................agcaccacaguauaccagUgc....................   1    1   Mm3
.......................................................................agcacAacaguauaccagggc....................   1    1   Mm3
.......................................................................agcaccacCguauaccagggc....................   1    1   Mm3
.......................................................................agcaccacaguauaccagggc....................   67   0   Mm3
.......................................................................agcaccacaguauaccagggcU...................   5    1   Mm3
```

```
                                   Star                            Mature
uaucagauaauggagaaaauugcauggcuuuguccccugguauccagugguugcucuuggggacaugaccaagcaccacaguauaccagggcauaugcaugcuucuagauuc
.......................................................................agcaccacaguauaccagggca...................   2      0   Mm3
........................................................................gcaccacaguauaccagggc....................   6      0   Mm3
........................................................................gcaccacaguauaccagggcU...................   1      1   Mm3
.........................................................................caccacaguauaccagggc....................   6      0   Mm3
.........................................................................caccacaguauaccagggcU...................   1      1   Mm3
..........................................................................accacaguauaccagggc....................   14     0   Mm3

...................................ccugguauccagugguugcucuugg....................................................   2      0   Fm3
..................................................................gaUcaagcaccacaguauaccagggc....................   2      1   Fm3
.....................................................................caagcaccacaguauaccaggg.....................   4      0   Fm3
.....................................................................caagcaccacaguGuaccagggc....................   1      1   Fm3
.....................................................................Uaagcaccacaguauaccagggc....................   2      1   Fm3
.....................................................................caagcaccacaguauaccagggc....................   18     0   Fm3
......................................................................aagcacUacaguauaccag.......................   1      1   Fm3
......................................................................aagcaccacaguauaccGg.......................   1      1   Fm3
......................................................................aagcaccacaguauaccag.......................   63     0   Fm3
......................................................................aagcaccacaguauaccagg......................   151    0   Fm3
......................................................................aagcaccacaguauacUagg......................   1      1   Fm3
......................................................................aagcaccacaguauacAagg......................   2      1   Fm3
......................................................................aagcaccacaguauaccagU......................   6      1   Fm3
......................................................................Uagcaccacaguauaccagg......................   1      1   Fm3
......................................................................aagcacGacaguauaccaggg.....................   1      1   Fm3
......................................................................aagcaccacaguauaccaggg.....................   521    0   Fm3
......................................................................aagcaccacaguauaccGggg.....................   3      1   Fm3
......................................................................aagcaccacaguauacGaggg.....................   2      1   Fm3
......................................................................aagcaccacaguauaccaggU.....................   7      1   Fm3
......................................................................aagcaccacaguauacUaggg.....................   1      1   Fm3
......................................................................aagcaccacaguauaccaggC.....................   3      1   Fm3
......................................................................Nagcaccacaguauaccaggg.....................   1      1   Fm3
......................................................................aagcaccacaguauaccUggg.....................   2      1   Fm3
......................................................................aagcaccacaguauacAaggg.....................   1      1   Fm3
......................................................................aagcaAcacaguauaccagggc....................   1      1   Fm3
......................................................................aagcaccacaguauaccaggCc....................   6      1   Fm3
......................................................................aagcaccacagGauaccagggc....................   2      1   Fm3
......................................................................aagcaccacaguauaccagAgc....................   6      1   Fm3
......................................................................aagcacGacaguauaccagggc....................   6      1   Fm3
......................................................................aagcaccacaguauaccagggc....................   37490  0   Fm3
......................................................................aagcaccacaguUuaccagggc....................   4      1   Fm3
......................................................................aagcaccacaguauaccagggU....................   85     1   Fm3
......................................................................aagcaccacaguaAaccagggc....................   6      1   Fm3
......................................................................aagGaccacaguauaccagggc....................   7      1   Fm3
......................................................................aagcaccacaguaCaccagggc....................   6      1   Fm3
......................................................................aaUcaccacaguauaccagggc....................   6      1   Fm3
......................................................................aagcaNcacaguauaccagggc....................   9      1   Fm3
......................................................................aagcacNacaguauaccagggc....................   5      1   Fm3
......................................................................Gagcaccacaguauaccagggc....................   6      1   Fm3
......................................................................aagcCccacaguauaccagggc....................   5      1   Fm3
......................................................................aagcaccacGguauaccagggc....................   29     1   Fm3
......................................................................aagcaccacaguauGccagggc....................   7      1   Fm3
......................................................................aagcaccUcaguauaccagggc....................   10     1   Fm3
......................................................................aCgcaccacaguauaccagggc....................   5      1   Fm3
......................................................................aagcaGcacaguauaccagggc....................   3      1   Fm3
......................................................................aagcaccacaguauaccagUgc....................   11     1   Fm3
......................................................................aagcaccacaguGuaccagggc....................   2      1   Fm3
......................................................................aagAaccacaguauaccagggc....................   8      1   Fm3
......................................................................aagcaccacaguauacGagggc....................   52     1   Fm3
......................................................................aagcacUacaguauaccagggc....................   19     1   Fm3
......................................................................aagcaccacaguauaccaggAc....................   4      1   Fm3
......................................................................aagcaccacagAauaccagggc....................   5      1   Fm3
......................................................................aagcaccacaguauaccagggA....................   42     1   Fm3
......................................................................aagcaccacCguauaccagggc....................   1      1   Fm3
......................................................................aagcGccacaguauaccagggc....................   4      1   Fm3
......................................................................aagcaccacaguauaccagggG....................   2      1   Fm3
......................................................................aaCcaccacaguauaccagggc....................   5      1   Fm3
......................................................................aagcaccacaguauaccGgggc....................   117    1   Fm3
......................................................................aagcaccacaguauaAcagggc....................   2      1   Fm3
......................................................................aagcaccacaguauaccaAggc....................   2      1   Fm3
......................................................................Nagcaccacaguauaccagggc....................   23     1   Fm3
......................................................................aagcaccacaguauaccCgggc....................   116    1   Fm3
......................................................................aagcaccacaguauacUagggc....................   137    1   Fm3
```

Star Mature

```
uaucagauaauggagaaaauugcauggcuuuguccccugguauccagugguugcucuuggggacaugaccaagcaccacaguauaccagggcauaugcaugcuucuagauuc
......................................................................aagcaccacaguauUccagggc....................   2     1    Fm3
......................................................................aagcaccaAaguauaccagggc....................   3     1    Fm3
......................................................................aagcaccacaguauaccagCgc....................   2     1    Fm3
......................................................................aagcaccacaguauaUcagggc....................   14    1    Fm3
......................................................................aagcaccGcaguauaccagggc....................   10    1    Fm3
......................................................................aUgcaccacaguauaccagggc....................   4     1    Fm3
......................................................................aaAcaccacaguauaccagggc....................   9     1    Fm3
......................................................................aagcaccacaAuauaccagggc....................   55    1    Fm3
......................................................................aagcaccCcaguauaccagggc....................   1     1    Fm3
......................................................................aagcacAacaguauaccagggc....................   3     1    Fm3
......................................................................aagcUccacaguauaccagggc....................   3     1    Fm3
......................................................................aagUaccacaguauaccagggc....................   4     1    Fm3
......................................................................Uagcaccacaguauaccagggc....................   28    1    Fm3
......................................................................aagcaUcacaguauaccagggc....................   11    1    Fm3
......................................................................aagcaccacaCuauaccagggc....................   37    1    Fm3
......................................................................aagcaccacaguauaccaggUc....................   8     1    Fm3
......................................................................aagcaccacUguauaccagggc....................   5     1    Fm3
......................................................................aagcaccaUaguauaccagggc....................   33    1    Fm3
......................................................................aagcaccacaguauacAagggc....................   182   1    Fm3
......................................................................aagcaccacaguCuaccagggc....................   2     1    Fm3
......................................................................aagcaccacaguaGaccagggc....................   4     1    Fm3
......................................................................aagcaccacaguauaccaUggc....................   21    1    Fm3
......................................................................aagcaccacagCauaccagggc....................   5     1    Fm3
......................................................................aGgcaccacaguauaccagggc....................   6     1    Fm3
......................................................................aagcaccacaguauaccUgggc....................   118   1    Fm3
......................................................................aagcaccacaUuauaccagggc....................   30    1    Fm3
......................................................................aagcaccacaguauaccagggca...................   105   0    Fm3
......................................................................aagcaccacaguauaccagggcU...................   224   1    Fm3
......................................................................aagcaccaUaguauaccagggca...................   8     1    Fm3
......................................................................aagcaccacaguauaccagggcC...................   3     1    Fm3
......................................................................aagcaccacaguauacUagggca...................   2     1    Fm3
......................................................................aagcaccaGaguauaccagggcau..................   6     1    Fm3
......................................................................aagcaccacaguauaccagggUau..................   1     1    Fm3
......................................................................aagcaccacaguauaccagggcau..................   6     0    Fm3
.......................................................................agcaccacaguauaccaCggc....................   1     1    Fm3
.......................................................................agcaccacaguauaccaggUc....................   1     1    Fm3
.......................................................................agcaccacagGauaccagggc....................   1     1    Fm3
.......................................................................agcaccacaguauaccCgggc....................   1     1    Fm3
.......................................................................agcaccacaguauaccaAggc....................   1     1    Fm3
.......................................................................agcaccacagAauaccagggc....................   1     1    Fm3
.......................................................................agcaccacagCauaccagggc....................   1     1    Fm3
.......................................................................aUcaccacaguauaccagggc....................   3     1    Fm3
.......................................................................agcaccacaguauaccagUgc....................   1     1    Fm3
.......................................................................agcaccacaguauaccagggc....................   109   0    Fm3
.......................................................................agcaccacaguauaccagggcU...................   1     1    Fm3
........................................................................gcaccacaguauaccagggc....................   1     0    Fm3
.........................................................................caccacaguauaccagggc....................   1     0    Fm3
..........................................................................accacaguauaccagggc....................   11    0    Fm3
...........................................................................ccacaguauaccagggcU...................   1     1    Fm3

...................................ccugguauccagugguugcucuugg....................................................   9     0    Mm1
......................................................................aagcaccacaguauaccaggg.....................   169   0    Mm1
......................................................................aagcaccacaCuauaccaggg.....................   1     1    Mm1
......................................................................aagcaccacaguauacAaggg.....................   2     1    Mm1
......................................................................aagcaccacaguauaccCggg.....................   1     1    Mm1
......................................................................aagcaccacaguauaccaggC.....................   1     1    Mm1
......................................................................aagcacAacaguauaccaggg.....................   1     1    Mm1
......................................................................aagcaccacaguauaccaggCc....................   2     1    Mm1
......................................................................Nagcaccacaguauaccagggc....................   6     1    Mm1
......................................................................aagcaccCcaguauaccagggc....................   1     1    Mm1
......................................................................aagcaccacaguauaccagggA....................   1     1    Mm1
......................................................................aagcaccacaguauaccagggU....................   4     1    Mm1
......................................................................aCgcaccacaguauaccagggc....................   1     1    Mm1
......................................................................aagcaccacaguauaccaggAc....................   1     1    Mm1
......................................................................aagUaccacaguauaccagggc....................   1     1    Mm1
......................................................................Uagcaccacaguauaccagggc....................   7     1    Mm1
......................................................................aagAaccacaguauaccagggc....................   1     1    Mm1
......................................................................aagcaccacagAauaccagggc....................   2     1    Mm1
......................................................................aagcaccacaguauaccaCggc....................   1     1    Mm1
......................................................................aagcaccacaguauaAcagggc....................   3     1    Mm1
```

```
                                   Star                              Mature
uaucagauaauggagaaaauugcauggcuuuguccccugguauccagugguugcucuuggggacaugaccaagcaccacaguauaccagggcauaugcaugcuucuagauuc
......................................................................aagcaccacaguauaccaAggc....................   1      1   Mm1
......................................................................aagcUccacaguauaccagggc....................   1      1   Mm1
......................................................................aagcaccacaguauaccagCgc....................   2      1   Mm1
......................................................................aagcaccacaguauaccagAgc....................   3      1   Mm1
......................................................................aaUcaccacaguauaccagggc....................   2      1   Mm1
......................................................................aagcaccacaguaCaccagggc....................   1      1   Mm1
......................................................................aagcaccacagGauaccagggc....................   1      1   Mm1
......................................................................aagcaNcacaguauaccagggc....................   1      1   Mm1
......................................................................aUgcaccacaguauaccagggc....................   2      1   Mm1
......................................................................aagcaccacaguauacAagggc....................   42     1   Mm1
......................................................................aaAcaccacaguauaccagggc....................   3      1   Mm1
......................................................................aagcGccacaguauaccagggc....................   1      1   Mm1
......................................................................aagcaccaUaguauaccagggc....................   5      1   Mm1
......................................................................aagcCccacaguauaccagggc....................   2      1   Mm1
......................................................................aagcaccacaguauaccagggc....................   11780  0   Mm1
......................................................................aagcaccaAaguauaccagggc....................   1      1   Mm1
......................................................................aagcaccacaguauaccUgggc....................   36     1   Mm1
......................................................................aagcaccacaguauGccagggc....................   1      1   Mm1
......................................................................aGgcaccacaguauaccagggc....................   3      1   Mm1
......................................................................aagcaUcacaguauaccagggc....................   2      1   Mm1
......................................................................aagcaccacaguauacUagggc....................   32     1   Mm1
......................................................................aagcaccacaguauaccaggUc....................   2      1   Mm1
......................................................................aagcacAacaguauaccagggc....................   2      1   Mm1
......................................................................aagcaccacaguUuaccagggc....................   2      1   Mm1
......................................................................Gagcaccacaguauaccagggc....................   1      1   Mm1
......................................................................aagcaccacaguauaccagUgc....................   6      1   Mm1
......................................................................aagcaccacaguauaccagggG....................   2      1   Mm1
......................................................................aagcaccacaguauacGagggc....................   23     1   Mm1
......................................................................aagcacUacaguauaccagggc....................   4      1   Mm1
......................................................................aagcaccacaguauaUcagggc....................   1      1   Mm1
......................................................................aagcaccUcaguauaccagggc....................   1      1   Mm1
......................................................................aagcaGcacaguauaccagggc....................   1      1   Mm1
......................................................................aagcaccacaUuauaccagggc....................   7      1   Mm1
......................................................................aagcaccacaAuauaccagggc....................   20     1   Mm1
......................................................................Cagcaccacaguauaccagggc....................   1      1   Mm1
......................................................................aagcaccacaguauaccGgggc....................   36     1   Mm1
......................................................................aagcacNacaguauaccagggc....................   1      1   Mm1
......................................................................aagcaccacaCuauaccagggc....................   8      1   Mm1
......................................................................aagcaccacaguauaccCgggc....................   42     1   Mm1
......................................................................aagcaccacGguauaccagggc....................   10     1   Mm1
......................................................................aagcaccacaguaAaccagggc....................   1      1   Mm1
......................................................................aagcaccacaguaGaccagggc....................   1      1   Mm1
......................................................................aagcaccacaguauaccagggcU...................   83     1   Mm1
......................................................................aagcaccacaguauaccagggca...................   11     0   Mm1
.......................................................................agcaccacaguauaccagggc....................   21     0   Mm1

...................................ccugguauccagugguugcu.........................................................   1      0   Mm2
...................................ccugguauccagugguugcucuu......................................................   1      0   Mm2
...................................ccugguauccagugguugcucuug.....................................................   5      0   Mm2
...................................ccugguauccaguggUGgcucuug.....................................................   1      1   Mm2
...................................ccugguauccagugguugcucuugg....................................................   22     0   Mm2
...................................ccugguauccaguggUCgcucuugg....................................................   1      1   Mm2
....................................cugguauccagugguugcucuugg....................................................   1      0   Mm2
......................................gguauccagugguugcucuugg....................................................   1      0   Mm2
.....................................................................caagcaccacaguauaccaggg.....................   1      0   Mm2
.....................................................................caagcaccacaguauaGcagggc....................   1      1   Mm2
.....................................................................Aaagcaccacaguauaccagggc....................   2      1   Mm2
.....................................................................caagcaccacaguauacAagggc....................   3      1   Mm2
.....................................................................caagcaccacaguauacGagggc....................   1      1   Mm2
.....................................................................caagcaccacaguauaccagggc....................   51     0   Mm2
.....................................................................caagcaccacaguauaccagggcU...................   2      1   Mm2
......................................................................aagcaccacaguauaccag.......................   7      0   Mm2
......................................................................aagcaccacaguauaccagg......................   46     0   Mm2
......................................................................aagcaccacaguauaccGgg......................   1      1   Mm2
......................................................................aagcaccacaCuauaccaggg.....................   1      1   Mm2
......................................................................aagcaccacGguauaccaggg.....................   1      1   Mm2
......................................................................Uagcaccacaguauaccaggg.....................   1      1   Mm2
......................................................................aagcaccacaguauaccaggg.....................   280    0   Mm2
......................................................................aagcaccacaguauaccaggC.....................   7      1   Mm2
......................................................................aagcaccacaguauaccGggg.....................   1      1   Mm2
```

```
                                   Star                                  Mature
uaucagauaauggagaaaauugcauggcuuugucccugguauccagugguugcucuuggggacaugaccaagcaccacaguauaccagggcauaugcaugcuucuagauuc
......................................................................aagcaccacaAuauaccaggg.....................   1       1   Mm2
......................................................................aagcUccacaguauaccaggg.....................   1       1   Mm2
......................................................................aagcaccacaguauacAaggg.....................   1       1   Mm2
......................................................................aagcaccacaguauaccCggg.....................   3       1   Mm2
......................................................................aagcaccacaguauaccagggA....................   18      1   Mm2
......................................................................aagcaccaUaguauaccagggc....................   43      1   Mm2
......................................................................aagcaccacaguauaccUgggc....................   285     1   Mm2
......................................................................aagcaccGcaguauaccagggc....................   12      1   Mm2
......................................................................aaUcaccacaguauaccagggc....................   27      1   Mm2
......................................................................aagUaccacaguauaccagggc....................   12      1   Mm2
......................................................................aaAcaccacaguauaccagggc....................   19      1   Mm2
......................................................................aagcaccacaUuauaccagggc....................   64      1   Mm2
......................................................................aagcaccacaguauUccagggc....................   14      1   Mm2
......................................................................Nagcaccacaguauaccagggc....................   59      1   Mm2
......................................................................aagcaccacagGauaccagggc....................   10      1   Mm2
......................................................................aagAaccacaguauaccagggc....................   19      1   Mm2
......................................................................aagcaccacagCauaccagggc....................   8       1   Mm2
......................................................................Cagcaccacaguauaccagggc....................   7       1   Mm2
......................................................................aagcaccacaguauaccagggU....................   134     1   Mm2
......................................................................aagcacNacaguauaccagggc....................   7       1   Mm2
......................................................................aagcaNcacaguauaccagggc....................   11      1   Mm2
......................................................................aagcaccaAaguauaccagggc....................   9       1   Mm2
......................................................................aagcaccacaguauaccaggAc....................   19      1   Mm2
......................................................................aagcaGcacaguauaccagggc....................   3       1   Mm2
......................................................................Gagcaccacaguauaccagggc....................   23      1   Mm2
......................................................................aagcaAcacaguauaccagggc....................   2       1   Mm2
......................................................................aagcaUcacaguauaccagggc....................   14      1   Mm2
......................................................................aUgcaccacaguauaccagggc....................   14      1   Mm2
......................................................................aagcaccacaguauaUcagggc....................   14      1   Mm2
......................................................................aagcaccacaguGuaccagggc....................   10      1   Mm2
......................................................................aagcaccacagAauaccagggc....................   9       1   Mm2
......................................................................aagcaccCcaguauaccagggc....................   7       1   Mm2
......................................................................aagcaccacaguauaccagggG....................   14      1   Mm2
......................................................................aagcaccacaguauaccagCgc....................   7       1   Mm2
......................................................................aagcaccacaguaGaccagggc....................   9       1   Mm2
......................................................................aagcacAacaguauaccagggc....................   4       1   Mm2
......................................................................aagcaccacUguauaccagggc....................   13      1   Mm2
......................................................................aagcaccacaguauaccaCggc....................   6       1   Mm2
......................................................................aagcaccacaguauCccagggc....................   4       1   Mm2
......................................................................aagcaccacaguauaccaUggc....................   14      1   Mm2
......................................................................aagcacUacaguauaccagggc....................   43      1   Mm2
......................................................................aagcaccacaguaCaccagggc....................   14      1   Mm2
......................................................................aagcaccacGguauaccagggc....................   59      1   Mm2
......................................................................aagcaccacaguauaccagAgc....................   15      1   Mm2
......................................................................aGgcaccacaguauaccagggc....................   20      1   Mm2
......................................................................aagcaccacaguauaccaAggc....................   7       1   Mm2
......................................................................aagcaccacaguauaccGgggc....................   301     1   Mm2
......................................................................aaNcaccacaguauaccagggc....................   4       1   Mm2
......................................................................aagcaccacaguUuaccagggc....................   4       1   Mm2
......................................................................aagcaccacaguauacAagggc....................   500     1   Mm2
......................................................................aagcUccacaguauaccagggc....................   16      1   Mm2
......................................................................aagcaccacaguauacGagggc....................   154     1   Mm2
......................................................................aCgcaccacaguauaccagggc....................   7       1   Mm2
......................................................................aagcaccacaguauaccaggCc....................   17      1   Mm2
......................................................................aagcaccUcaguauaccagggc....................   21      1   Mm2
......................................................................aagcaccacCguauaccagggc....................   4       1   Mm2
......................................................................aagcCccacaguauaccagggc....................   6       1   Mm2
......................................................................aagcaccacaguauacUagggc....................   332     1   Mm2
......................................................................aagcaccacaguCuaccagggc....................   4       1   Mm2
......................................................................aagcaccacaguauGccagggc....................   29      1   Mm2
......................................................................aaCcaccacaguauaccagggc....................   9       1   Mm2
......................................................................aagcaccacaguauaGcagggc....................   2       1   Mm2
......................................................................Uagcaccacaguauaccagggc....................   79      1   Mm2
......................................................................aagcaccacaguauaccagggc....................   103434  0   Mm2
......................................................................aagcaccacaguaAaccagggc....................   15      1   Mm2
......................................................................aagcaccacaguauaccCgggc....................   306     1   Mm2
......................................................................aagGaccacaguauaccagggc....................   7       1   Mm2
......................................................................aagcaccacaAuauaccagggc....................   154     1   Mm2
......................................................................aagcaccacaCuauaccagggc....................   88      1   Mm2
......................................................................aagcacGacaguauaccagggc....................   3       1   Mm2
```

Star Mature

```
uaucagauaauggagaaaauugcauggcuuuguccccugguauccagugguugcucuuggggacaugaccaagcaccacaguauaccagggcauaugcaugcuucuagauuc
......................................................................aagcaccacaguauaccaggUc....................  20    1  Mm2
......................................................................aagcGccacaguauaccagggc....................  17    1  Mm2
......................................................................aagcaccacaguauaAcagggc....................  6     1  Mm2
......................................................................aagcaccacaguauaccagUgc....................  18    1  Mm2
......................................................................aagcacUacaguauaccagggca...................  1     1  Mm2
......................................................................aagcaccacaguauaccUgggca...................  1     1  Mm2
......................................................................aagcaccacaguauaccagggcC...................  17    1  Mm2
......................................................................aagcaccacaguauaccagggcG...................  14    1  Mm2
......................................................................aagcaccacaguauaccagggca...................  110   0  Mm2
......................................................................aagcaccacaguauacUagggca...................  1     1  Mm2
......................................................................aagcaccacaguauaccagggcU...................  809   1  Mm2
......................................................................aagcaccacaguauaccagggcCu..................  6     1  Mm2
......................................................................aagcaccacaguauaccagggUau..................  1     1  Mm2
......................................................................aagcaccacaguauaccagggcUu..................  5     1  Mm2
......................................................................aagcaccaGaguauaccagggcau..................  20    1  Mm2
......................................................................aagcaccacaguauaccagggcaA..................  2     1  Mm2
......................................................................aagcaccacaguauaccagggcau..................  5     0  Mm2
.......................................................................agcaccacaguauaccagggc....................  196   0  Mm2
.......................................................................agcaccacaguGuaccagggc....................  1     1  Mm2
.......................................................................agGaccacaguauaccagggc....................  1     1  Mm2
.......................................................................agcaccacaguauaccaCggc....................  1     1  Mm2
.......................................................................agcaccacaguauaccCgggc....................  1     1  Mm2
.......................................................................agcaccacaguauGccagggc....................  1     1  Mm2
.......................................................................Ngcaccacaguauaccagggc....................  1     1  Mm2
.......................................................................agcaccacaguauaccGgggc....................  1     1  Mm2
.......................................................................agcaccaAaguauaccagggc....................  1     1  Mm2
.......................................................................agcaccacaguauaccagggca...................  2     0  Mm2
.......................................................................agcaccacaguauaccagggcU...................  6     1  Mm2
........................................................................gcaccacGguauaccagggc....................  1     1  Mm2
........................................................................gcaccacaguauaccagggc....................  2     0  Mm2
.........................................................................caccacaguauaccagggc....................  5     0  Mm2
..........................................................................accacaguauaccagggc....................  1     0  Mm2

...................................ccugguauccagugguugcucuugg....................................................  9     0  Fm2
.....................................................................caagcaccacaguauaccagggc....................  3     0  Fm2
......................................................................aagcaccacaguauaccagg......................  4     0  Fm2
......................................................................aagcaccacaguauaccaggg.....................  9     0  Fm2
......................................................................aagcaccacaguauaUcagggc....................  15    1  Fm2
......................................................................aCgcaccacaguauaccagggc....................  2     1  Fm2
......................................................................aagcaccacagAauaccagggc....................  1     1  Fm2
......................................................................aagcaccacaguauacUagggc....................  43    1  Fm2
......................................................................aagcaccacaguaAaccagggc....................  1     1  Fm2
......................................................................Uagcaccacaguauaccagggc....................  6     1  Fm2
......................................................................aagAaccacaguauaccagggc....................  2     1  Fm2
......................................................................aagcaccacaCuauaccagggc....................  7     1  Fm2
......................................................................aagcaccacaguauacGagggc....................  14    1  Fm2
......................................................................aagcaccacaguaCaccagggc....................  4     1  Fm2
......................................................................aagcaccacaguauaccagggG....................  1     1  Fm2
......................................................................aagcaccacaguauaccGgggc....................  28    1  Fm2
......................................................................aagcaccacaguauUccagggc....................  1     1  Fm2
......................................................................aagcaccacGguauaccagggc....................  3     1  Fm2
......................................................................aagcaccacaguauaccagUgc....................  1     1  Fm2
......................................................................aagcaUcacaguauaccagggc....................  2     1  Fm2
......................................................................aagcaccacaguauaccCgggc....................  32    1  Fm2
......................................................................aGgcaccacaguauaccagggc....................  2     1  Fm2
......................................................................aagcaccacaguauaccaggUc....................  1     1  Fm2
......................................................................aagcaccacaguauacAagggc....................  29    1  Fm2
......................................................................aagcaccacaguauaccagggc....................  9753  0  Fm2
......................................................................aagcaNcacaguauaccagggc....................  3     1  Fm2
......................................................................Gagcaccacaguauaccagggc....................  1     1  Fm2
......................................................................Nagcaccacaguauaccagggc....................  4     1  Fm2
......................................................................aaUcaccacaguauaccagggc....................  5     1  Fm2
......................................................................aagcaccacaguauaccUgggc....................  28    1  Fm2
......................................................................aagcaccacaguauaccagAgc....................  1     1  Fm2
......................................................................aagcacUacaguauaccagggc....................  4     1  Fm2
......................................................................aagcGccacaguauaccagggc....................  2     1  Fm2
......................................................................aagcaccaAaguauaccagggc....................  1     1  Fm2
......................................................................aagcaccaUaguauaccagggc....................  14    1  Fm2
......................................................................aagcacGacaguauaccagggc....................  1     1  Fm2
......................................................................aagGaccacaguauaccagggc....................  2     1  Fm2
```

```
                                   Star                              Mature
uaucagauaauggagaaaauugcauggcuuuguccccugguauccagugguugcucuuggggacaugaccaagcaccacaguauaccagggcauaugcaugcuucuagauuc
......................................................................aagcUccacaguauaccagggc....................    1     1     Fm2
......................................................................aagcaccacaUuauaccagggc....................    9     1     Fm2
......................................................................aagcaccacCguauaccagggc....................    1     1     Fm2
......................................................................aagcacAacaguauaccagggc....................    1     1     Fm2
......................................................................aagcaccacaguauaccaUggc....................    4     1     Fm2
......................................................................aaAcaccacaguauaccagggc....................    3     1     Fm2
......................................................................aagcaccacaguauGccagggc....................    3     1     Fm2
......................................................................aagcaccacaAuauaccagggc....................    10    1     Fm2
......................................................................aagcaccacaguauaccaggAc....................    2     1     Fm2
......................................................................aagcaccacaguauaccagggU....................    21    1     Fm2
......................................................................aagcaccacaguauaccagggA....................    1     1     Fm2
......................................................................aagcaccacaguauaAcagggc....................    2     1     Fm2
......................................................................aagcacUacaguauaccagggca...................    1     1     Fm2
......................................................................aagcaccacaguauaccagggca...................    31    0     Fm2
......................................................................aagcaccacaguauaccagggcU...................    59    1     Fm2
......................................................................aagcaccacaguauacAagggca...................    1     1     Fm2
......................................................................aagcaccacaguauaccagggcaA..................    20    1     Fm2
......................................................................aagcaccacaguauaccagggcCu..................    8     1     Fm2
......................................................................aagcaccaGaguauaccagggcau..................    11    1     Fm2
.......................................................................agcaccacaguauaccagggc....................    29    0     Fm2

...................................ccugguauccagugguugcucuug.....................................................    2     0     Fm1
...................................ccugguauccagugguugcucuuggggacaugacc..........................................    7     0     Fm1
.....................................................................caagcaccacGguauaccaggg.....................    1     1     Fm1
.....................................................................caagcaccacaguauaccaggg.....................    3     0     Fm1
......................................................................aagcaccacaguauUccagg......................    1     1     Fm1
......................................................................aagcaccacaguauaccagg......................    11    0     Fm1
......................................................................aagcaccacaguauaccaggg.....................    35    0     Fm1
......................................................................aagcaccacaguauaccaggC.....................    4     1     Fm1
......................................................................aagcaccacaguauaccaggAc....................    2     1     Fm1
......................................................................aagcacUacaguauaccagggc....................    9     1     Fm1
......................................................................aaAcaccacaguauaccagggc....................    4     1     Fm1
......................................................................aagcaccacaguauaccaggUc....................    1     1     Fm1
......................................................................aagcaccaUaguauaccagggc....................    11    1     Fm1
......................................................................aagcaccacaguauaccagCgc....................    4     1     Fm1
......................................................................aagcaccacaguauacGagggc....................    32    1     Fm1
......................................................................Nagcaccacaguauaccagggc....................    19    1     Fm1
......................................................................aagcacAacaguauaccagggc....................    1     1     Fm1
......................................................................Uagcaccacaguauaccagggc....................    13    1     Fm1
......................................................................aagcaccacaguauUccagggc....................    1     1     Fm1
......................................................................aagcaccacaAuauaccagggc....................    31    1     Fm1
......................................................................aagcaccacaguauaccaggCc....................    2     1     Fm1
......................................................................aagcaccacaUuauaccagggc....................    9     1     Fm1
......................................................................aUgcaccacaguauaccagggc....................    2     1     Fm1
......................................................................aagcaccacaguauaccagAgc....................    1     1     Fm1
......................................................................aagcaccacaguaCaccagggc....................    2     1     Fm1
......................................................................aagcaccGcaguauaccagggc....................    7     1     Fm1
......................................................................aagUaccacaguauaccagggc....................    2     1     Fm1
......................................................................aagcaccacaguauaccagggU....................    48    1     Fm1
......................................................................aagcaNcacaguauaccagggc....................    4     1     Fm1
......................................................................aagcaccacagAauaccagggc....................    2     1     Fm1
......................................................................aagcCccacaguauaccagggc....................    2     1     Fm1
......................................................................aagcaccacaguauacUagggc....................    55    1     Fm1
......................................................................aaUcaccacaguauaccagggc....................    6     1     Fm1
......................................................................Gagcaccacaguauaccagggc....................    4     1     Fm1
......................................................................aagcaccacagCauaccagggc....................    2     1     Fm1
......................................................................aagcaccacUguauaccagggc....................    5     1     Fm1
......................................................................aagcaccacaguauaccagggG....................    2     1     Fm1
......................................................................aagcaccacaguauaccCgggc....................    62    1     Fm1
......................................................................aCgcaccacaguauaccagggc....................    1     1     Fm1
......................................................................aagcaUcacaguauaccagggc....................    2     1     Fm1
......................................................................aagcaccacaguauaccaCggc....................    1     1     Fm1
......................................................................aagcaccacaguauaccaUggc....................    7     1     Fm1
......................................................................aagcaccacaguauaUcagggc....................    5     1     Fm1
......................................................................aagcaccacaguauaccagggA....................    4     1     Fm1
......................................................................aagcaccacaguaAaccagggc....................    4     1     Fm1
......................................................................aagcGccacaguauaccagggc....................    2     1     Fm1
......................................................................aagcaccacCguauaccagggc....................    1     1     Fm1
......................................................................aagcaccacaguauaccUgggc....................    62    1     Fm1
......................................................................aGgcaccacaguauaccagggc....................    2     1     Fm1
```

```
                                                   Star                              Mature
uaucagauaauggagaaaauugcauggcuuugucccugguauccagugguugcucuuggggacaugaccaagcaccacaguauaccagggcauaugcaugcuucuagauuc
......................................................................aagcaccacaguauaccGgggc....................   55      1     Fm1
......................................................................aagAaccacaguauaccagggc....................   2       1     Fm1
......................................................................aagcaccacaCuauaccagggc....................   11      1     Fm1
......................................................................aagcaccacaguauGccagggc....................   1       1     Fm1
......................................................................Cagcaccacaguauaccagggc....................   2       1     Fm1
......................................................................aagcaccUcaguauaccagggc....................   2       1     Fm1
......................................................................aagcaccacaguauaccagUgc....................   2       1     Fm1
......................................................................aagcUccacaguauaccagggc....................   2       1     Fm1
......................................................................aagcaccacaguUuaccagggc....................   4       1     Fm1
......................................................................aagcaccacaguauaccagggc....................   19185   0     Fm1
......................................................................aagcaccacGguauaccagggc....................   13      1     Fm1
......................................................................aagcaccacaguaGaccagggc....................   4       1     Fm1
......................................................................aagcaccacaguauacAagggc....................   68      1     Fm1
......................................................................aagcaccacaUuauaccagggca...................   1       1     Fm1
......................................................................aagcaccacaguauaccagggcC...................   7       1     Fm1
......................................................................aagcaccacaguauaccGgggca...................   1       1     Fm1
......................................................................aagcaccacaguauaccagggca...................   53      0     Fm1
......................................................................aagcaccacaguauaccagggAa...................   4       1     Fm1
......................................................................aagcaccacaguauacUagggca...................   1       1     Fm1
......................................................................aagcaccacaguauaccagggcU...................   237     1     Fm1
......................................................................aagcaccacaguauaccagggcG...................   7       1     Fm1
......................................................................aagcaccacaguauaccagggcUu..................   5       1     Fm1
......................................................................aagcaccaGaguauaccagggcau..................   29      1     Fm1
......................................................................aagcaccacaguauaccagggcCu..................   2       1     Fm1
......................................................................aagcaccacaguauaccagggcau..................   5       0     Fm1
......................................................................aagcaccacaguauaccagggcauU.................   2       1     Fm1
......................................................................aagcaccaGaguauaccagggcaua.................   2       1     Fm1
.......................................................................agcaccacaguauaccagggc....................   28      0     Fm1
.......................................................................agcaccacaguauaccagggcU...................   2       1     Fm1
```
